# Phase transformation-driven artificial muscle mimics the multifunctionality of avian wing muscle

**DOI:** 10.1098/rsif.2020.1042

**Published:** 2021-11-03

**Authors:** Pedro B. C. Leal, Marcela Cabral-Seanez, Vikram B. Baliga, Douglas L. Altshuler, Darren J. Hartl

**Affiliations:** ^1^ Department of Aerospace Engineering, Texas A&M University, College Station, TX 77843, USA; ^2^ Department of Zoology, University of British Columbia, Vancouver, British Columbia, Canada V6T 1Z4

**Keywords:** shape memory alloys, biomimetics, bipedal locomotion, work-loops, multifunctional, muscles

## Abstract

Skeletal muscle provides a compact solution for performing multiple tasks under diverse operational conditions, a capability lacking in many current engineered systems. Here, we evaluate if shape memory alloy (SMA) components can serve as artificial muscles with tunable mechanical performance. We experimentally impose cyclic stimuli, electric and mechanical, to an SMA wire and demonstrate that this material can mimic the response of the avian humerotriceps, a skeletal muscle that acts in the dynamic control of wing shapes. We next numerically evaluate the feasibility of using SMA springs as artificial leg muscles for a bipedal walking robot. Altering the phase offset between mechanical and electrical stimuli was sufficient for both synthetic and natural muscle to shift between actuation, braking and spring-like behaviour.

## 1. Introduction

**Figure 1.**
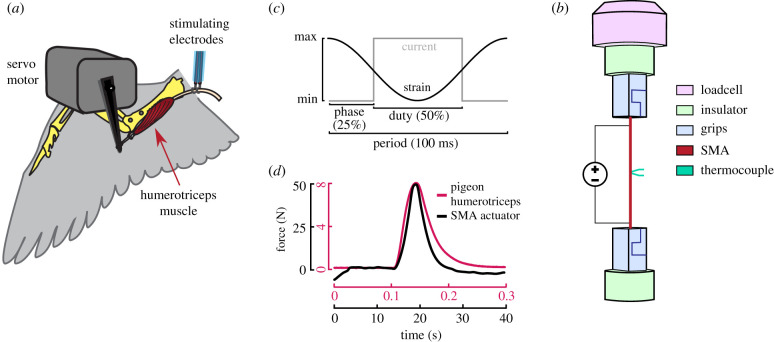
Set-up for experiments with humerotriceps muscles of pigeons and SMA actuators. (*a*) The humerotriceps muscle originates on the head of the humerus and, for work loop measurements, the insertion was attached to a servo motor arm. The dorsal branch of the brachial nerve was draped over two hooked electrodes and received electrical impulses to stimulate the muscle [7] (adapted from Theriault *et al.* [7], with permission). (*b*) The engineering actuator consists of an equiatomic nickel–titanium wire attached to a loading frame responsible for the strain stimuli via insulated grips on each side. (*c*) Schematic of the electric and mechanical inputs for work-loop studies. (*d*) Isometric twitch of the pigeon humerotriceps muscle [7] and block force of a nitinol wire reveal similar asymmetry in force onset versus force offset.

In traditional engineered systems, actuators, brakes and structural members represent distinct components. By contrast, when an animal moves through its environment, all such functions can be performed by a single integrated musculoskeletal system [1]. Dynamic variation in functional demands is common to all forms of animal locomotion, but is an extreme feature of powered flight [2]. Birds dynamically adjust the shape and position of their wings to tailor performance to atmospheric conditions such as high winds [3,4], and while capturing prey and gliding [5]. Insight into ‘wing morphing’ adaptations that accommodate changing conditions is a major design target for improving aircraft performance. An important mechanism believed to be responsible for shape change in the wing is the elbow motion [6] driven by two triceps muscles, the humerotriceps and scapulotriceps. Theriault *et al.* [7] determined that in the humerotriceps muscles of pigeons (*Columba livia*), the timing of neural stimuli relative to the muscle length cycle (hereafter ‘phase offset’) affected the production of mechanical power (figure 1*a*). Electrically stimulating the muscle just before attaining peak length resulted in actuation, whereas nearly all other phase offsets resulted in either dissipation (a brake) or low hysteresis (a spring). This observed multi-functional capability of a single wing muscle raises the question of whether human-designed systems can be designed to shift among functional roles with minimal changes to applied stimuli.

Bar-Cohen postulated that the design gap between natural and engineered systems [8] can be bridged by the use of smart materials such as shape memory alloys (SMAs). SMA components, usually formed from nickel and titanium alloys (nitinol), generate and recover large strains (displacements) under large stresses (forces) and dissipate energy when undergoing phase transformation between austenite and martensite [9]. Depending on the initial state and thermomechanical loading, different phenomena can be observed. When not transforming, SMA components are linearly elastic, and can be used as structural members. However, if sufficiently mechanically loaded, an initially austenitic nitinol specimen will transform and deform toward a microstructural configuration of oriented martensite variants in a macroscopically nonlinear fashion and then fully recover the austenitic configuration when unloaded. This is known as the pseudoelastic effect, and it can be highly dissipative. A specimen in an oriented martensite state can also be heated until it transforms into austenite and recovers large strains under high loads in a nonlinear manner known as the shape memory effect. SMA actuators can generate positive mechanical work given the appropriate timings of supplied thermal energy [10]. Collectively, these features of SMA point to its potential use as an artificial muscle [11] with tunable mechanical performance [12], but it is unknown whether SMA can replicate the breadth of multifunctional capability shown by natural skeletal muscle, especially given cyclic stimuli.

Because the functional role of skeletal muscle has been shown to be dependent on phase offset [13–15], we sought to determine how SMA systems respond to phase offset of supplied thermal stimuli. We first performed experimental studies to measure the mechanics inherent to an SMA system. This revealed that an SMA system can have negative, neutral and positive work-loops in a fashion that emulates those shown by skeletal muscle, namely the humerotriceps muscles of pigeons [7]. For the purposes of both clear illustration and motivation of the biomimetics and active materials communities, we next performed numerical investigations considering a relatively simple locomotive response. The goal is to demonstrate how potential engineering applications might benefit from the carefully tuned force dynamics of SMA components, such tuning being commonly employed in natural systems [16].

## 2. Methods

### 2.1. Shape memory alloy artificial muscle set-up

All experiments use the set-up depicted in figure 1 and are here described. Two stimuli, an electrical and a mechanical, were imposed on the SMA wire. The *electrical stimuli* in the form of voltage were directly applied to the wire to increase temperature via Joule heating; cooling is provided via natural convection. Type-*K* thermocouples are attached to the nitinol wire with a silver thermal paste to measure temperature, and two ABS 3D-printed connectors were used to electrically insulate the load frame from the nitinol wire. The *mechanical stimuli* in the form of displacement were controlled through an MTS Insight tensile testing machine and force was measured by a 100 N loadcell. The strain is defined as cosine wave with the same frequency as the electric pulse wave, which is further defined by duty, phase offset and amplitude. For each work-loop, five cycles are imposed, and only the last three are studied to remove outliers.

### 2.2. Bipedal compass model

**Figure 2.**
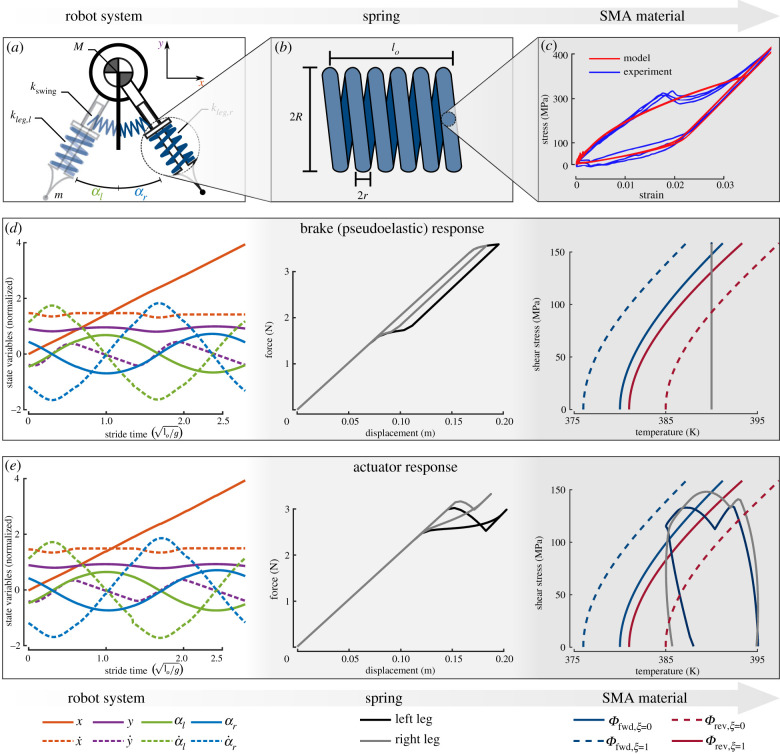
Modelling framework of an adaptive bipedal robot from systems to material level and examples. Concept analysis requires the coupling of the following models: (*a*) a dynamic model for the transient bipedal locomotion for a variable stiffness spring (parameters: *k*_*leg*,*l*_, *k*_*leg*,*r*_, *k*_*swing*_ and *M*), (*b*) a phenomenological model coupling local strain to spring displacements (parameters: *r*, *R* and *N*), and (*c*) a one-dimensional constitutive model for the thermomechanical response of the SMA material. (*d*) The resultant states for systems- (location and velocity of the robot), actuator- (force and displacement) and material-levels (stress, strain and temperature) for a constant temperature (*T* = 390 K), and (*e*) for a cyclic thermal stimulus (*T*_*o*_ = 387.8571, Δ*T* = 2.8571, *θ*_*r*_ = 0.5143, *θ*_*l*_ = 0.9000, and frequency of $0.7429/\sqrt{l_{o}g}\;\mathrm{Hz}$).

The primary goal of this work is to quantitatively compare and contrast the cyclic work loop behaviour of natural muscle with an SMA tensile actuator. Doing so in the context of locomotion allows additional comparison between natural and engineered systems, where future exploration by researchers and designers is encouraged. To this end, we choose a simple and openly available locomotion model that considers the interplay of gravity, inertia and elasticity; it is a spring-loaded inverted pendulum model that approximates running gaits [17,18] and that allows full reproducibility by the reader. Specifically, we used the model by Remy and coworkers, as shown in figure 2*a*, which has extensively been used for various gait problems [19–21]. Incorporated into this is the high-fidelity SMA constitutive model for the leg components [9,22] (see §2.4) in spring form (see §2.3).

The spring-loaded inverted pendulum model of the compass robot consists of a main body with mass *M*, two legs with mass *m* and stiffnesses *k*_*leg*,*l*_ and *k*_*leg*,*r*_, and an elastic torsional spring between both legs with stiffness *k*_swing_ (omitted from the figure). Leg mass is neglected, and each leg has an SMA compression spring element of length, *l*_*l*_ and *l*_*r*_, and undeformed length *l*_*o*_. The system is defined by the coordinates (*x*, *y*) of the centre of mass as well as the pitch angles *α*_*l*_ and *α*_*r*_ for the left and right legs. As typical for bipedal locomotion, all results are normalized based on body size dimensions [23]. For a non-dissipative leg spring, an angular frequency *ω*_swing_ of the leg motion is guaranteed by defining the torsional spring stiffness as

2.1 $$k_{\mathrm{swing}}=\omega_{\mathrm{swing}}^{2}ml_{o}^{2}.$$

During locomotion, at most one foot at a time can be in contact with the ground, and no-slip conditions are assumed. During contact, the angle and length of each leg are defined through $\alpha_{i}=\arctan\left[(s_{i}-x)/y\right]$ and $l_{i}=\sqrt{{\left(x-s_{i}\right)}^{2}+y^{2}}$, where *s*_*i*_ is the *x*-component of the spring length, and the reaction forces are

2.2 $$\begin{array}{rl} F_{x} & =k_{leg,l}(l_{l}-l_{o})\sin\alpha_{l}+k_{leg,r}(l_{r}-l_{o})\sin\alpha_{r}\\ \mathrm{and}\;F_{y} & =k_{leg,l}(l_{l}-l_{o})\cos\alpha_{l}+k_{leg,r}(l_{r}-l_{o})\cos\alpha_{r}. \end{array}\}$$

However, when not in contact, the leg is assumed to be at the rest length *l*_*o*_. The transition in between contact and no-contact is defined by a series of events (e.g. take-off and touchdown) as defined by Gan *et al.* [19].

The trajectory of the robot centre of mass in the state space was not imposed to understand the dynamic interplay of all acting physical phenomena and the role that they play in generating efficient mechanics observed in gait [24]. Contrary to the experiments in figures 1 and 4, the strain and stress paths were not known *a priori* and were calculated as the robot advanced, with the following underlying ordinary differential equations [19–21]:

2.3 $$\begin{array}{rl} \ddot{x} & =F_{x}/M,\\ \ddot{y} & =F_{y}/M-g,\\ {\ddot{\alpha }}_{l} & =\frac{1}{l_{o}^{2}m}[-\ddot{x}ml_{o}\cos\alpha_{l}-(g+\ddot{y})ml_{o}\sin\alpha_{l}-k_{\mathrm{swing}}\alpha_{l}]\\ \mathrm{and}\;{\ddot{\alpha }}_{r} & =\frac{1}{l_{o}^{2}m}[-\ddot{x}ml_{o}\cos\alpha_{r}-(g+\ddot{y})ml_{o}\sin\alpha_{r}-k_{\mathrm{swing}}\alpha_{r}]. \end{array}\}$$

In the state space, the four governing equations can be constructed in the canonical form of eight first-order differential equations that are fully defined by $\left\{x,\dot{x},y,\dot{y},\alpha_{l},{\dot{\alpha }}_{l},\alpha_{r},{\dot{\alpha }}_{r}\right\}$ and integrated via a fourth-order Runge–Kutta scheme, calculating all state variables as shown in the first column of figure 2*d*,*e*. The initial conditions were taken from the work of Gan *et al.* [19], and the resultant elastic gait is classified as a ‘running forward symmetrical single stance’ [19]. The robot parameters and initial conditions are provided in table 1. Spring stiffnesses are calculated for each instance of time for the instantaneous thermomechanical states, as is explained in the following sections.

**Table 1.** List of parameters used for numerical simulations.

compass-gait parameters	*M*	10 kg
	*g*	9.81 kg m s^−2^
	*l*_*o*_	0.526 m
	*ω*_swing_	7
spring parameters	*r*	3.4 mm
	*C*	3
	*N*	113
SMA constitutive model parameters	*G*_*A*_	14.4 GPa
	*G*_*M*_	34.2 GPa
	*M*_*s*_	380 K
	*M*_*f*_	376 K
	*A*_*s*_	381 K
	*A*_*f*_	385 K
	*C*_*A*_	7.20 MPa K^−1^
	*C*_*M*_	7.95 MPa K^−1^
	*H*_min_	0
	*H*_max_	0.055
	*κ*	4.68 GPa^−1^
	*τ*_*crit*_	0 Pa
	*n*_1_	0.18
	*n*_2_	0.18
	*n*_3_	0.15
	*n*_4_	0.29
compass-gait initial conditions (non-dimensional)	$x_{o}$	0
	${\dot{x}}_{o}$	1.4709
	*y*_*o*_	0.9053
	${\dot{y}}_{o}$	−0.3840
	*α*_*l*,*o*_	−0.4528
	${\dot{\alpha }}_{l,o}$	1.1412
	*α*_*r*,*o*_	0.4387
	${\dot{\alpha }}_{r,o}$	−1.1684
SMA initial conditions (both springs)	*ξ*_*o*_	0
	$\epsilon_{o}^{t}$	0
	*σ*_*o*_	0
	*ε*_*o*_	0

### 2.3. Spring model

Nitinol is only able to generate at most 6% recoverable extensional strain [25] and would not provide sufficient compliance if formed into the most common wire/rod configurations [26]. An SMA spring was utilized among many possible options for balancing force against displacement [27]. The nitinol components along an SMA spring are undergoing pure shear that is not uniform along the radius of the wire. For engineering purposes, the constitutive model for the cross-section of the SMA wire is defined based on an effective shear stress $\bar{\tau }$ and strain $\bar{\gamma }$ via

2.4 $$\bar{\tau }=G\bar{\gamma },$$

where *G* is shear modulus. Force *F* and displacement *u* at the systems level are associated with the local $\bar{\tau }$ and $\bar{\gamma }$ of the SMA springs through [27]

2.5 $$\begin{array}{cc} & F=\frac{2\pi r^{2}}{3C}\bar{\tau }\\ \mathrm{and}\;\;\;\; & u=\frac{8\pi NC^{2}r}{3}\bar{\gamma }, \end{array}\}$$

where *C* is the spring index, *r* is the wire radius and *N* is the number of coils; alongside the undeformed length *l*_*o*_, these four parameters fully define the spring geometry as shown in figure 2*b*; the selected parameter values are depicted in table 1. Examples of spring force and displacement when functioning as a brake or an actuator are provided in the second column of figure 2*d*,*e*.

### 2.4. Shape memory alloy model

The thermomechanical behaviour of SMAs can be described by constitutive models that establish a phenomenological description of these alloys. Herein, the model elaborated by Lagoudas *et al.* [9] is modified for shear strain and briefly introduced in the sequence. The model considers three external state variables: effective shear stress $\bar{\tau }$, effective shear strain $\bar{\gamma }$ and absolute temperature *T*. Two internal state variables are also considered: effective inelastic transformation strain ${\bar{\gamma }}^{t}$ (i.e. caused by detwinning) and martensitic volume fraction *ξ*. The model follows the Gibbs free energy principle, and temperature and total strain are known. For this paper, temperature is known while stress is calculated through linear and angular momentum conservation. Additive decomposition is assumed by considering elastic and inelastic contributions; hence for a lumped-parameter model [9]

2.6 $$\bar{\gamma }=\left[\frac{1}{G_{A}}+\xi \left(\frac{1}{G_{M}}-\frac{1}{G_{A}}\right)\right]\bar{\tau }+{\bar{\gamma }}^{t},$$

where *G*_*A*_ and *G*_*M*_ are the shear modulus for austenitic and martensitic phases.

Crystal structure transformation only takes place at specific thermechanical states according to the transformation direction, *forwards* from austenite (*ξ* = 0) to martensite (*ξ* = 1) or *reverse* from martensite to austenite. The region where transformation is possible is defined through transformation functions $\Phi_{\mathrm{rev}}$ and $\Phi_{\mathrm{fwd}}$ as depicted in the third column in figure 2*d*,*e*. The relation between the evolution of inelastic transformation strain ${\bar{\gamma }}^{t}$ and evolution of martensitic volume fraction is given as

2.7 $${\dot{\bar{\gamma }}}^{t}=\dot{\;\xi }\left\{ \begin{array}{cc} \mathrm{sgn}(\bar{\tau })H^{\mathrm{cur}}(|\bar{\tau }|) & \dot{\;\xi }>0,\\ {\bar{\gamma }}^{t-r}/\xi^{r} & \dot{\;\xi }\leq 0, \end{array}\right.$$

where *H*^cur^ is the current transformation strain, $\bar{\gamma }{\;}^{t-r}$ is the effective transformation strain at transformation reversal, and *ξ*^*r*^ is the martensitic volume fraction at transformation reversal.

The magnitude of transformation strain generated during full martensite transformation is captured by the scalar-valued function $H^{\mathrm{cur}}(|\bar{\tau }|)$. For trained materials, *H*^cur^ is as follows:

2.8 $$H^{\mathrm{cur}}(|\bar{\tau }|)=\left\{ \begin{array}{cc} H_{\min} & |\bar{\tau }|>\tau_{\mathrm{crit}},\\ H_{\min}+(H_{\max}-H_{\min})(1-e^{-\kappa (|\bar{\tau }|-\tau_{\mathrm{crit}})}) & |\bar{\tau }|\leq \tau_{\mathrm{crit}}, \end{array}\right.$$

where *H*_min_, *H*_max_, *κ* and *τ*_*crit*_ are material parameters. Other parameters considered in the model are: the transformation temperatures (*A*_*s*_, *A*_*f*_, *M*_*s*_ and *M*_*f*_) that determine where transformation occurs when no stress is applied, the slope of the transformation surfaces (*C*_*A*_ and *C*_*M*_), and the hardening parameters (*n*_1_, *n*_2_, *n*_3_ and *n*_4_) that determine the smoothness in the transition between transformation and thermoelastic loading. An in-depth description of each variable is provided by Lagoudas *et al.* [9], and selected parameter values are provided in table 1.

## 3. Results

### 3.1. Artificial versus natural muscles

The experimental set-up for the investigation of artificial muscles includes an equiatomic nitinol wire subject to tensile loading via an MTS Insight tensile testing machine and to electrical stimuli via Joule heating as depicted in figure 1*b*. This set-up is capable of timed mechanical and electrical stimuli to the SMA wire, reproducing the studied stimuli on humerotriceps muscles [7].

We first assessed the transient response of SMA systems to electrical stimuli (figure 1*d*). Similar to the isometric twitch investigation performed by Theriault *et al.* [7], we subjected SMA specimens to a block force test; here, the specimen lengths were held constant while a pulse of electrical stimulus was applied. This initial assessment indicated clear similarities between nitinol wire and pigeon triceps muscle responses although in different time scales, particularly in the asymmetry in force onset versus offset, which then motivated the investigation of other shared functionalities.

In cyclic operation (figure 1*c*), a stimulated muscle contracts and produces force, which can either provide positive work by shortening or can provide negative work as it resists lengthening [13]. Muscle performance in animals is influenced by muscle length, velocity, stimulation intensity and timing, and imposed force change during a cycle [2]. *In situ* work-loops, as depicted in figure 4*a*,*b*, give an estimate of the *in vivo* output work for biological muscles. Varying the phase offset between the mechanical and electrical cyclic stimuli alters the net work produced. A positive work cycle, counterclockwise in the force–displacement (stress–strain) space, corresponds to the muscle functioning as an actuator. Negative work, clockwise in stress–strain space, equates to a dissipative brake. Although not considered explicitly herein, the muscle can also result in zero net work cycles similar to a spring if its stress–strain response is non-hysteretic. Therefore, the humerotriceps can function as a brake, actuator, or a spring with variable compliance depending on activation properties [7].

**Figure 3.**
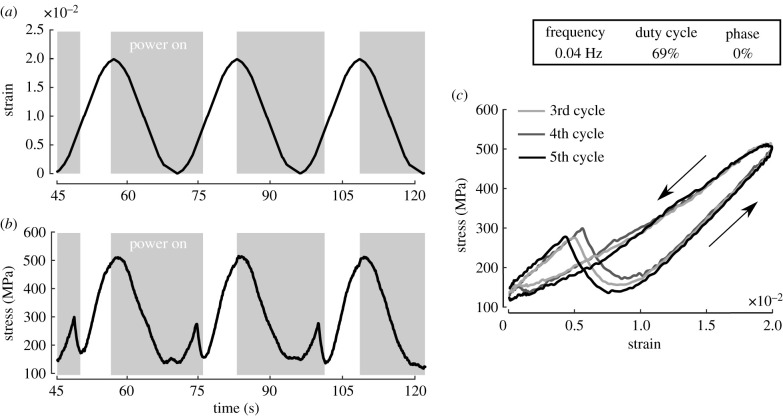
Cyclic response of SMA wire for 69% duty cycle, 0.04 Hz, and 0% phase. Similar data are available for all experiments in the electronic supplementary material. (*a*) The implied mechanical and electrical stimuli. (*b*) Resultant stress as a function of time. (*c*) Stress and strain evolution for the last three cycles. The counterclockwise loop directionality characterizes energy added to the system.

The exploration of work-loops in the SMA wire expanded upon the previous block force testing by instead imposing a cyclic strain with amplitude of 0.02 within each of three frequency treatments. Timed Joule heating represented the imposed actuation stimulus while cooling occurred via forced convection. The stimuli frequencies studied were based on the explored frequencies by Theriault *et al.* [7] and adjusted to take in consideration the intrinsically different time scales between the natural and artificial muscle responses (cf. figure 1*d*). Considering this set-up, the influences of frequency (29, 40 and 48 mHz), duty cycle (50% and 69%), and phase offset (−25%, 0%, 25% and 50%) were experimentally explored. The mechanical results for one of the evaluated cases are depicted in figure 3 to demonstrate the cyclic response as a function of time. Similar to Theriault *et al.* [7], the first two cycles are neglected to consider results with reproducible heat transfer (i.e. the same temperature at the end of each cycle). The majority of experiments lead to reproducible cycles, whereas the case here depicted has the most variability. The applied stimuli, figure 3*a*, result in the stress output depicted in figure 3*b*,*c*. The net work for the depicted work-loop is positive, characterizing an actuator.

**Figure 4.**
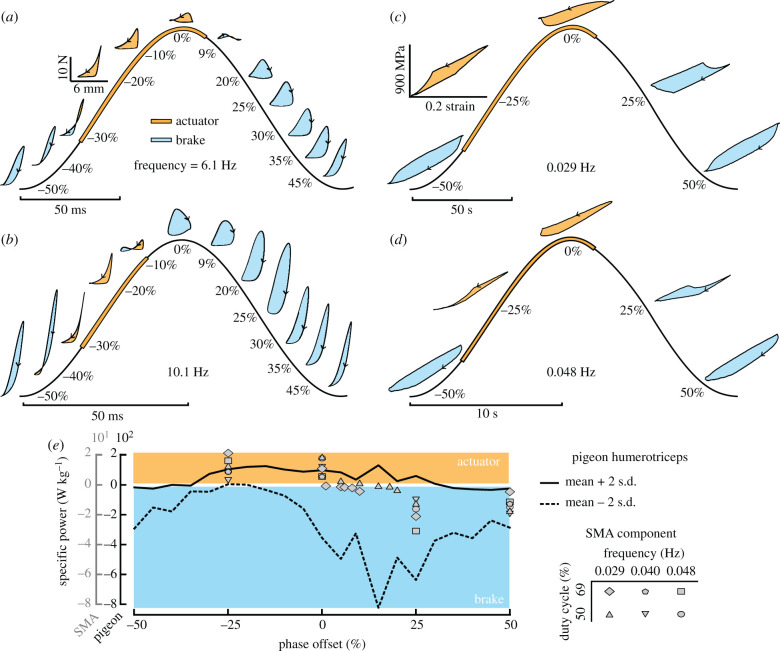
*In situ* work loops reveal the humerotriceps muscles of pigeons and SMA actuators are both capable of multifunctional roles through phase offset of stimuli. (*a*) Work-loops for pigeon humerotriceps specimen at 6.1 Hz and 50% stimulus duty cycle [7] and (*b*) at 10.1 Hz and 50% stimulus duty cycle [7]. (*c*) Work-loops for SMA component at 29 mHz and 50% stimulus duty cycle and (*d*) at 48 mHz and 50% stimulus duty cycle. Percentages indicate phase offset of stimulus (relative to peak strain). Arrows on work loop traces depict the direction of the loops, with counterclockwise loop directionality indicating that the net work is positive (orange fill); clockwise directionality indicates net work is negative (blue fill). (*e*) Power output for the pigeon humerotriceps and the SMA actuator in response to phase offset.

It was expected that the SMA wire would behave as a brake for most explored cycles given the dissipative nature of many mechanic cycles previously explored [28]. This behaviour was observed for phase offsets 25% and 50% for the work-loop cycles depicted in figure 4*c*,*d*. However, if the electrical stimuli were timed to phase offsets between −25% and 0%, the work-loops evolve in a counterclockwise manner, corresponding to positive net work output. Therefore, it is possible to use the same SMA wire as a brake, structural member, and actuator solely as a function of thermal stimuli.

The overall similarity in work loop shape and direction motivated us to determine how strong the effects of phase offset are on power output in both natural and artificial materials. We evaluated the nonlinear monotonic relationship between stimulus parameters and net work using the Spearman rank correlation factor *r*_*s*_ [29].^1^ Similar to natural muscle systems, the phase offset of the thermal stimuli on SMA is strongly correlated to the resultant power output with *r*_*s*_ = −0.75. The effects of frequency and duty cycles were not significant for the explored ranges^2^ with respective Spearman rank correlations of −0.03 and −0.04. This predominant dependency on stimuli phase offset is similar to that observed by natural systems [7]. For the pigeon humerotriceps, the Spearman values for phase offset, frequency, and duty cycle were −0.49, −0.30 and −0.01. Considering all the experimental results for natural and artificial muscles, the same trends are noticeable regarding specific power. Figure 4*e* depicts the data for varying frequencies, duty cycles, and phase offsets solely as a function of phase offset and specific power. Both systems are mostly dependent on the phase offset and act as an actuator for approximately the same phase ranges, a similarity that motivated us to further explore the use of SMA components as artificial muscles.

Despite the similar responses to electrical stimuli, the driving mechanism for each material is intrinsically different. The electrical stimulation is converted to heat via the Joule effect for SMA material, leading to solid-to-solid phase transformation [25]. While for muscles, neuronal stimulus elicits a depolarization that ultimately increases intracelluar calcium availability, leading to muscle fibre contraction [30]. Because of this fundamental difference, actuation frequency and functional fatigue (i.e. fatigue regarding generated strain) also differ. Regarding cycling, SMA response is dominated by the ‘slow’ process of convection-driven cooling as phase transformation depends on heat transfer. By contrast, skeletal muscle responses are a function of the attachment/detachment rates of actin and myosin filaments within the cell [31]. In either case, relaxation is the slower process in both materials (cf. figure 1*d*). Regarding functional fatigue, this occurs in an SMA component because of micro-crack initiation, micro-crack propagation, martensite transformation induced damage, and others [32], while in muscles, fatigue is caused by a shortage of fuels (e.g. glycogen) within the muscle fibre, accumulation of metabolites, enervation of the nervous system and others [33]. Despite key differences in actuation frequency and functional fatigue, SMA and skeletal muscle show similar work loop trends, which motivates the exploration of SMA components as artificial muscles in engineering applications.

### 3.2. Robotics application to multifunctional shape memory alloy components

All results hitherto indicate that SMA components enable tailored structural properties in cyclic applications. Previous studies have aspired to develop various potential engineered systems mimicking biological counterparts and specifically have implemented SMA components in biomimetic jellyfish [34,35], birds [26,36], earthworms [37], human-like anatomical systems [38], and others. However, previous studies did not explore the multifunctional potential of SMA components as inspired by natural musculoskeletal systems. Here, we examine the prospect of SMA for non-smooth systems [39], specifically bipedal locomotion [40]. This or similar locomotor modes may be of interest for bioinspired engineering because in animals it allows for the exploration of challenging habitats that are not currently accessible by wheeled and tracked vehicles [41]. Simple rigid bipedal robots can achieve some necessary gaits [42,43], but timed and tailored compliance plays an essential role in nature [44] by allowing energy storage and release as well as improving passive adaptability [45]. Here, we employ a simple and openly available model to demonstrate these effects.

**Figure 5.**
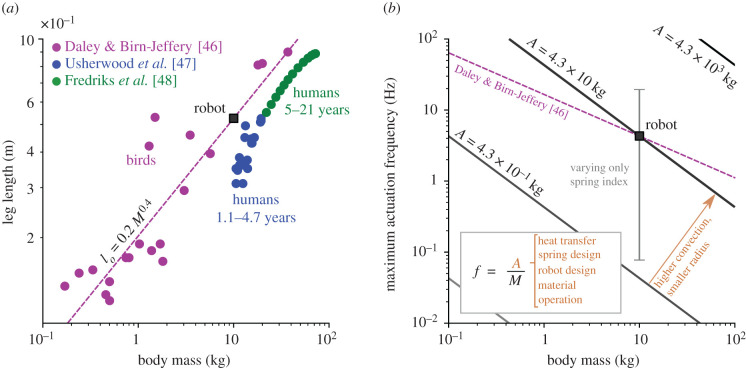
Scalability of the SMA-based robot in regards to dimensions, mass, and frequency. (*a*) Relation between leg length and body mass for: birds [46], 1.1–4.7-year-old humans [47], and 5–21-year-old humans [48]. Robot leg length is selected to satisfy the correlation from Daley and Birn-Jeffery, *l*_*o*_ = 0.2*M*^0.4^. Fredriks *et al.* did not provide information regarding the body mass of the studied population. Still the metric is estimated based on body mass index data for ages 5–19 provided by the World Health Organization. (*b*) Maximum actuation frequency of SMA spring relative to robot body mass for bipedal locomotion. The relation between both metrics is derived in appendix A.

While the compass gait model from Gan *et al.* [19] is non-dimensional, here we assume a reasonable but purely illustrative leg length and body mass of 0.526 m and 10 kg for calculating the mechanical state of the SMA wire cross section. The leg length of the 10 kg robot was selected based on the avian leg length to body mass correlation from the work of Daley & Birn-Jeffery [46] as shown in figure 5*a*. The used leg length is 50% above that of a child with equivalent body mass. For the selected dimensions, the SMA component can achieve actuation frequencies beyond the robot stride frequency with the proper combination of parameters as shown in figure 5*b*. The maximum actuation frequency is inversely proportional to the body mass times a coefficient *A* as derived in appendix A. As the body mass of the robot increases, the maximum actuation frequency decreases for the same coefficient *A*, corresponding to a constant set of parameters (e.g. heat transfer, spring design, operation and others). Parameters such as the spring index *C*, typically between 4 and 25, can significantly impact the maximum actuation frequency as shown by the grey line in figure 5*b*. The developed frequency–mass relation, equation (A7), and the Daley and Birn-Jeffery correlation can be combined to determine the maximum actuation frequency for the selected robot parameters as a function of mass as shown by the magenta line in figure 5*b*. Overall, SMA components can function for bipedal locomotion for the explored frequency, body mass and leg length domains.

Despite the merits of SMA bipedal locomotion, it is here used as a theoretical testing platform for tunable work-loops of artificial muscles. While researchers have shown that SMA-based legs can lead to dynamic actuation and untethered locomotion at biologically relevant speeds [49,50], there are engineering challenges that would have to be overcome. Electrically heating the SMA component minimizes auxiliary equipment but the additional weight of a cooling mechanism can be prohibitive [51]. For the design proposed, *A* = 4.3 × 10^1^ kg, the minimum required heat convection coefficient, *h* = 425 W (m^2^ K)^−1^, would be at the upper limit of what can be achieved with forced air convection and at the lower limit for forced liquid convection [52]. Therefore, it is feasible to use these traditional cooling mechanisms for untethered SMA-driven soft robots, as previously shown for air [53] and water [54]. Integration of such a cooling device could be challenging, but could be facilitated by using a unique heating/cooling solution such as liquid metal [55] or by using the device in a highly convective environment [56]. Moreover, for the same *A* coefficient, a robot with greater dimensions such as *l*_*o*_ = 5.26 m or at Moon’s gravity would have less stringent cooling requirements, *h* = 134 and *h* = 172 W (m^2^ K)^−1^, respectively. Therefore, full configurational specification of an SMA-based bipedal robot is technically feasible, but is out of the scope of this study. Practically speaking, it is expected that the discussions herein may be more impactful as engineers consider much smaller systems with SMA components operating at higher frequencies given only ambient convective cooling [50].

**Figure 6.**
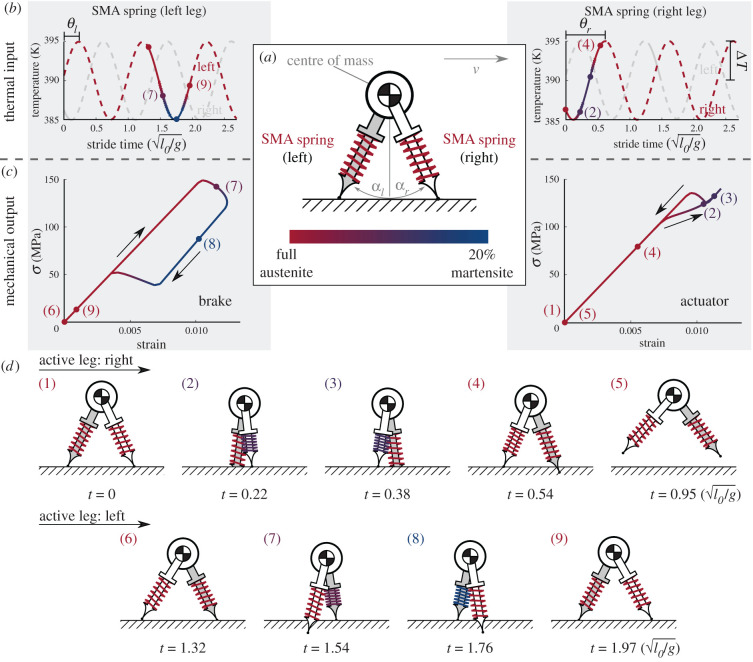
Example of how SMAs afford multifunctionality that can tune performance. (*a*) Graphical representation of a simplified bilocomotion model using SMA springs to either drive or add compliance to the system. (*b*) Thermal stimuli for SMA springs on right and left legs. (*c*) Mechanical output for each leg with the respective volume fractions depicted. During the first cycle of locomotion, the right leg functions as an actuator followed by the left leg that operates as a brake. The operation of each leg is dependent on applied thermal stimuli. (*d*) Various states of the transient locomotion of the robot. The mechanical output for each state is also denoted in (*c*).

In the field of robotics, sinusoidal stimuli can generate gaits that are similar to those found in nature (e.g. running of a cockroach [57]). Moreover, sine wave inputs have also been employed for natural muscles (e.g. angle extensors in wild turkeys [14]). Therefore, we used sinusoidal thermal stimuli for our robot prototype [14]. The sinusoidal temperature imposed on each leg has the same frequency, amplitude Δ*T*, and mean temperature but the phase offsets, *θ*_*r*_ and *θ*_*l*_, are different. A solution where the SMA components have opposite functions (i.e. actuator versus brake) is depicted in figure 6*b*,*c*. As the robot walks, the martensitic volume fraction *ξ* of the springs varies when in contact with the ground. All cases explored represent only partial transformation (ξ<20%), which is beneficial to the component fatigue life [58,59], and thus use only a fraction of the contracting capability of the artificial muscle (10%) similar to muscle fibres constrained by joints (20%) [12]. These results demonstrate that an SMA component can preserve or dissipate energy to achieve more natural gaits with a simple mechanism, not requiring the use of other components such as latches [60].

**Figure 7.**
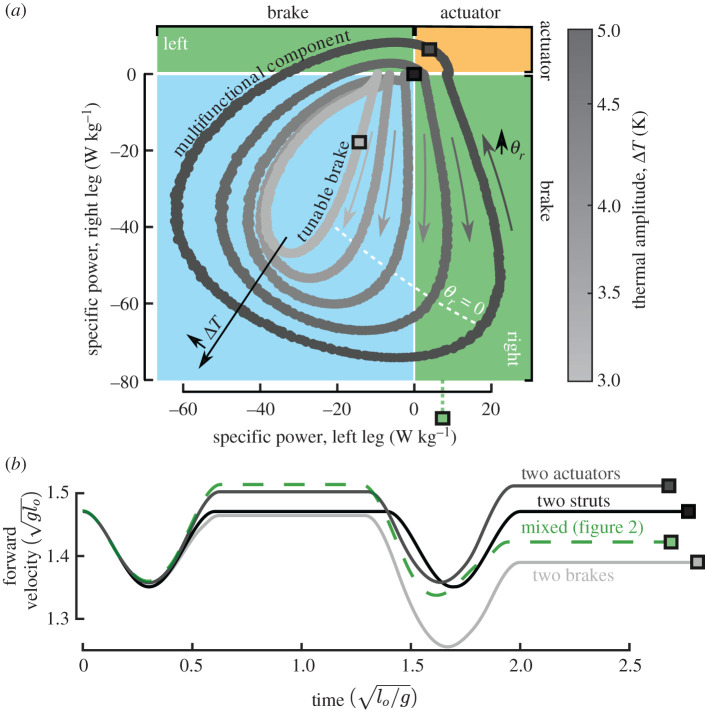
Operational tunability of SMA is a function of thermal amplitude and phase offset. (*a*) Influence of right leg phase offset *θ*_*r*_ and thermal amplitude Δ*T* on power output from each leg. Frequency, left phase offset and mean temperature are held constant at $\sqrt{g/l_{o}}$, 0, and 390 K. (*b*) Examples of how SMA power output affects robot horizontal velocity (e.g. two actuators increase velocity and two brakes decrease velocity).

The real value of using SMA components is the tuning capability [61] resulting from phase transformation; this is elucidated in figure 7. Changes in time-variant temperature stimuli can result in different features from those depicted in figure 6. If the thermal amplitude is fixed at Δ*T* = 5 K and only the right leg phase offset is varied, the compression springs operate as various combinations of brakes, structural members and actuators, as shown in figure 7*a*. Therefore, the thermal stimuli can be modified such that energy is provided to drive locomotion or dissipated based on environmental conditions (e.g. terrain and gust) or manoeuvre objectives (e.g. trajectory and velocity requirements). Some applications only require a dissipative functionality, but benefit from varying dissipation magnitude [61]. By decreasing the thermal stimuli amplitude, multiple tunable brake configurations are possible that are a function of a single parameter. A direct consequence of varying the net cyclic system energy is a decrease or increase of the robot instantaneous velocity, as depicted in figure 7*b*. Note that all results herein depicted (cf. figures 6 and 7) are for specific robot and spring specifications; more general design studies may identify other responses of interest.

**Figure 8.**
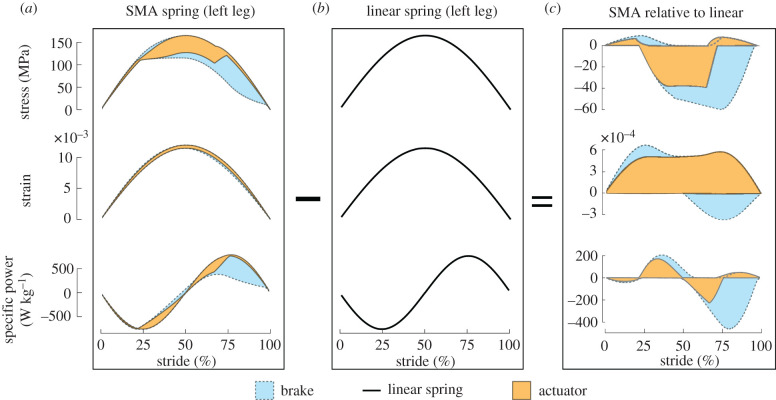
Mechanical response for all explored stimuli inputs (cf. figure 7) as a function of stride. All explored gaits have the same initial conditions for the left leg. (*a*) Stress, strain and specific power bounds for gaits classified by the SMA spring response as an actuator or a brake. (*b*) Mechanical response of the bipedal robot with a linear spring and Young’s modulus equivalent to *E*_*A*_. (*c*) Difference between modified gaits (i.e. actuator and brake) relative to the linear spring.

The obtained mechanical results in figure 7 can also be utilized to provide insight on how the mechanical states (i.e. stress, strain and power) change along with the stride and the reason why the SMA component acts as a brake or an actuator. The upper and lower bounds of all evaluated SMA responses are provided in figure 8*a* in a similar fashion as the bird gait study by Higham *et al.* [62]. While the actuator and brake strains are similar, the stress significantly differs at the end of the stride, from 70 to 95%. As a consequence, less power is generated at the end of the stride for a brake SMA component. Any stimuli combination will add or subtract energy throughout the stride regardless of whether the net work is positive or negative. Even for the case of a linear spring where the net work is zero, negative and positive instantaneous power is experienced as shown in figure 8*b*. Comparing the actuator and brake performances relative to the linear spring as shown in figure 8*c* provides further insight why some stimuli result in actuators. Actuator solutions do not decrease the strain and stress at the end of the cycle relative to the zero-work performance, resulting in more energy being added rather than subtracted as for most brakes. Therefore, the SMA spring can increase power at specific points of the stride provided timely stimulus is applied, leading to a narrow set of solutions with net positive work.

## 4. Discussion

That skeletal muscle can achieve multifunctionality, transitioning between actuator, brake or spring-like behaviour, via changes to stimulus phase has been well established [2,7,14,15]. Despite engineering efforts to mathematically characterize biological systems capable of this multifunctionality [30], no study had heretofore reproduced this feature for an engineered system with adaptive structures. We explored the capabilities of SMAs as artificial muscles based on the similarity of the mechanical response of phase transformation with muscle fibre phenomenology [12] (cf. figures 1 and 2). We observed, through experimental and numerical studies, highly similar trends in work and power output in response to phase offset by skeletal muscles and SMA-based artificial muscles (cf. figures 3 and 4). Both systems’ work-loops were highly sensitive to the phase offset of the electrical input, indicating that energy output can be tailored during operation by shifting stimulus phase.

**Figure 9.**
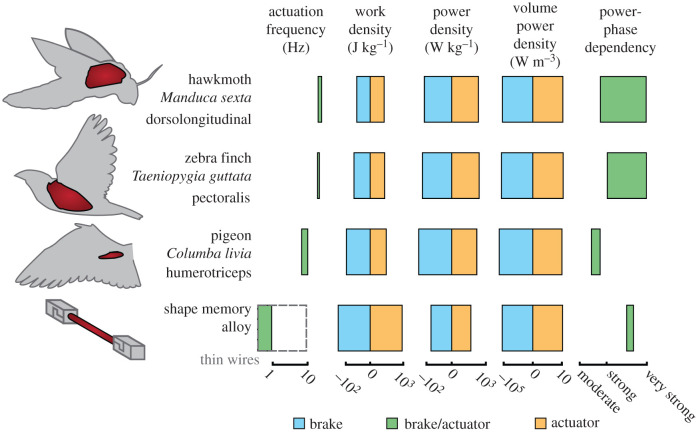
Muscles and SMAs exhibit similar capability for braking and actuation under different stimulus phases, despite substantial differences in actuation frequency and power phase dependency. We compare the performance of SMA, including from a previous study of thin wire SMA [50], with those found in previous studies of muscles that also examined the effects of phase offset to timed stimuli: hawkmoth dorsolongitudinal [63], zebra finch pectoralis [64] and pigeon humerotriceps [7] muscles. Hawkmoth frequency range attained from Willmot & Ellington [65]. To mitigate the influence of outliers, only the average of each cyclic experiment is considered. More details are provided in the electronic supplementary material.

Comparing the performance capabilities of skeletal muscle and SMA reveals these materials have similar capacity for braking and actuation under different stimulus phases, despite substantial differences in actuation frequency *as tested* (cf. figures 7 and 8). In figure 9, we provide a comparison of work-related properties of SMA and skeletal muscles that have been examined in the context of phase offset to timed stimuli: hawkmoth dorsolongitudinal [63], zebra finch pectoralis [64] and pigeon humerotriceps [7] muscles. Although the specific SMA specimens tested herein (i.e. in figures 3 and 4) operated at slower strain rates than is typical of skeletal muscle, thin-wire preparations of SMA [50] allow higher frequency oscillations that approach the operating frequencies of skeletal muscles. Note that frequency is a system performance metric that greatly increases in SMAs as component size decreases due to the increasing ratio of surface area to material volume and the associated dynamics of heat transfer. This is explicitly shown with the dashed grey box in figure 9, which compares the frequency range tested herein with the frequencies possible in thinner wires [50]. Work density is the product of actuation stress, actuation strain and the inverse of density; all three are material properties independent of component size and a broad range of specific work output is possible in these materials when compared with natural muscles. Power density is then the product of work density and cyclic frequency, the latter being strongly dependent on component size; higher frequencies and higher power densities are restricted to smaller, lower force systems. Furthermore, SMA shows as strong a dependency of tunability on phase shift as compared to the primary muscles that power flight in hawkmoths and birds, and a stronger relationship between power and phase compared to that in the pigeon’s humerotriceps. Collectively, each of these materials affords control authority over functional output, which can be tuned via adjustments to phase offset. What is especially promising is that SMAs show greater range of work per unit mass over studied ranges, indicating that SMA is a valuable material if adaptive changes to the magnitude of force or work production is the target of design.

Muscle constitutive behaviour plays a critical role in understanding animal locomotion [7] as well as developing efficient bipedal locomotion [44]. In our effort to explore the design domain of artificial muscles, SMA components are incorporated in a novel bipedal locomotion model as a stiffness-controlled spring element. The tunable spring compliance adds a mechanism that can increase or decrease the amount of energy delivered to or dissipated from locomotion, changing robot gait. This adaptivity is expected to benefit other applications beyond bipedal locomotion. As the work-loop response of the humerotriceps (cf. figure 4) is the same as that of other muscles used for flying, this multifunctional mechanism could aid the design of other bioinspired devices. It may provide biological insight regarding the design of more efficient engineering systems, while also providing engineering insight into movement and coordination in biological systems that cannot be easily directly measured (e.g. bird wings) [6].

## Endnotes

1 *r*_*s*_ ∈ [ − 1, 1], where negative values indicate an inversely proportional relationship. Correlations are qualified as ‘very weak’ (*r*_*s*_ ∈ ±[0.0, 0.2[), ‘weak’ (*r*_*s*_ ∈ ±[0.2, 0.4[), ‘moderate’ (*r*_*s*_ ∈ ±[0.4, 0.6[), ‘strong’ (*r*_*s*_ ∈ ±[0.6, 0.8[) and ‘very strong’ (*r*_*s*_ ∈ ±[0.8, 1.0[).

2 Higher values of frequency and duty cycles were not explored because they would lead to higher temperatures, and, as a result, the nitinol wire would predominantly act as an elastic structural component.

## Data accessibility

The data that support the findings of this study are available with the online version of this paper. A description of all the provided files can be found in the electronic supplementary material. The original compass gait model is provided by Gan *et al.* at Bitbucket (https://bitbucket.org/ganzhenyu/bipedalslipmodel_swingleg/src/master/) while the model utilized for this work is available at GitHub (https://github.com/leal26/gait).

## Appendix A. Derivation of actuation frequency to body mass ratio

A relation between the maximum actuation frequency of an SMA spring located at the robot leg and the body mass of the bipedal robot is derived based on conservation of linear momentum and energy. During the stride, the robot weight and ground reaction force, equivalent to the elastic spring force, counter each other with the maximum load on the SMA springs taking place at approximately 50% of the stride. In that instance, the elastic force is aligned with gravity, and the *conservation of linear momentum* considering the spring stiffness definition (equation (2.5)) and stretch *δ*_*l*_ = (*l*_*l*_ − *l*_*o*_)/*l*_*o*_ is

A 1 $$Mg=\frac{Gr}{4NC^{3}}\delta_{l}l_{o},$$

solving for the wire radius *r*:

A 2 $$r=\frac{4NMgC^{3}}{G\delta_{l}l_{o}}.$$

A second equation is derived from *conservation of energy* by making three assumptions. The maximum frequency of an SMA is dominated by the cooling period [55]. Considering that the heating period is at most an order of magnitude less than cooling, the heating phase is neglected for calculating actuation frequency. Moreover, heat contributions from phase transformations are neglected as all studied cases only had partial phase transformation. Finally, thermal expansion is neglected as the temperature ranges are not significant. Conservation of energy [66] considering the three assumptions reduces to governing equation to Newton’s cooling law. The solution to the equation for cooling a wire at initial temperature *T*_*i*_ to final temperature *T*_*f*_ at ambient temperature *T*_∞_ is [67]

A 3 $$T_{f}=(T_{i}-T_{\infty })e^{-(h/\rho c)\Delta t}+T_{\infty },$$

where *c* is the SMA heat capacity, Δ*t* is the time necessary to cool down the specimen and *h* is the heat transfer coefficient. *h* is a function of the wire radius, the Nusselt number *Nu* and the fluid thermal conductivity *k* such that

A 4 $$h=\frac{kNu}{2r}.$$

Solving Newton’s cooling law, equation (A 3), for Δ*t* and considering the definition of the convection coefficient, the actuation frequency is given by

A 5 $$f=\frac{1}{\Delta t}=-\frac{kNu}{2\rho cr}{\left[\ln\frac{T_{f}-T_{\infty }}{T_{i}-T_{\infty }}\right]}^{-1}.$$

Finally, the definition of radius from conservation of linear momentum, equation (A 2), is incorporated in the actuation frequency equation from conservation of energy to derive the following final relation:

A 6 $$f=\frac{A}{M},$$

where

A 7 $$A=-\frac{G}{8\rho cg}\frac{kNu\delta_{l}l_{o}}{NC^{3}}{\left[\ln\frac{T_{f}-T_{\infty }}{T_{i}-T_{\infty }}\right]}^{-1}.$$

The lower and upper bounds for coefficient *A* are 4 × 10^−7^ and 9 × 10^3^ kg for the typical ranges for each parameter.
